# Laryngopharyngeal Reflux

**DOI:** 10.1155/2012/926806

**Published:** 2012-09-06

**Authors:** Petros D. Karkos, Wolfgang Issing, Oliver Reichel, Marcus Hess

**Affiliations:** ^1^Department of Otolaryngology-Head Neck Surgery, Queen Alexandra Hospital, Portsmouth PO63LY, UK; ^2^Department of Otolaryngology, Aristotle university of Thessaloniki, Thessaloniki, Greece; ^3^Department of Otolaryngology-Head Neck Surgery, The Freeman Hospital, Newcastle Upon Tyne, UK; ^4^Department of Otolaryngology-Head Neck Surgery, Schwarzwald-Baar Hospital, Villingen-Schwenningen, Germany; ^5^Department of Otolaryngology-Head Neck Surgery, University Medical Center Hamburg-Eppendorf, Hamburg, Germany

Laryngopharyngeal reflux (LPR) is here to stay. Despite advances in the role of pepsin, better drugs, and less invasive tests, diagnosis is still difficult and is still in many places—based on empiric treatment rather than robust evidence.

In this special issue on LPR, we attempt to review some hot topics regarding this entity which still puzzles otolaryngologists all over the world.

The issue of chronic cough, postnasal drip, and reflux is quite a controversial topic and remains a therapeutic challenge. Chronic cough can be associated with many diseases that often overlap more than one medical specialty. A detailed assessment of the patient with chronic cough relies on a multidisciplinary approach and close cooperation between pulmonary medicine, gastroenterology, and otolaryngology. Gastroesophageal reflux (GERD) and postnasal drip syndrome account for a significant number of cases of chronic nonproductive cough seen in otolaryngology practice. Each may, alone or in combination, contribute to cough even when clinically silent and failure to recognise their contribution may lead to unsuccessful treatment. Many of these patients are notoriously difficult to diagnose and treat but the literature suggests that a systematic and thorough approach in a multidisciplinary setting can lead to successful diagnosis and treatment in the majority of patients.

The role of surgery in management of LPR is also a matter of debate in the literature. Results of laparoscopic fundoplication for the treatment of classic GERD are well established. In theory, surgery for persistent LPR after failure of medical treatment should be equally as effective, if we agree on the notion that LPR and GERD share common pathophysiology. Many studies have demonstrated symptomatic improvement after surgical fundoplication. However, current knowledge does not allow this conclusion. Large multicenter, randomized control trials are needed, focusing on diagnostic tools to improve selection criteria, presenting standard endpoints and long-term follow-up.

Eosinophilic esophagitis (EE) is a great reflux mimic and often presents with dysphagia, recurrent food bolus obstruction and GERD-like symptoms. Despite higher awareness, the literature suggests that EE remains a commonly misdiagnosed condition especially in the otolaryngology community. The treatment though differs than the LPR treatment and EE should be part of the differential diagnosis when faced with “difficult to treat LPR or GERD”. The introduction of Transnasal Esophagoscopy in the ENT office over the last decade has meant that increasingly more laryngologists become accustomed in recognising esophageal pathology including EE.

The presence of specific endoscopic laryngeal findings in patients with suspected LPR has been well documented in the literature. Because the first-line therapy for LPR is considered to be proton pump inhibitors, there are many studies comparing the endoscopic laryngeal findings before and after acid suppression therapy.

Finally, the role of pepsin and the fact that LPR is much more dependent on pepsin-mediated damage in the laryngeal and airway mucosa than on acid have been well described over the last few years. The crucial role of pepsin in LPR may, in turn, stimulate the development of drugs which specifically target this molecule. This may radically enhance our knowledge and management of this condition.

*Petros D. Karkos* *Petros D. Karkos* *Wolfgang Issing* *Wolfgang Issing* *Oliver Reichel* *Oliver Reichel* *Marcus Hess* *Marcus Hess*

